# Echocardiographic epicardial fat thickness and immature granulocyte are novel inflammatory predictors of acute ischemic stroke: a prospective study

**DOI:** 10.1590/1516-3180.2021.0461.R1.16082021

**Published:** 2022-04-11

**Authors:** Mustafa Korkut, Fatih Selvi, Cihan Bedel

**Affiliations:** I MD. Emergency Physician, Department of Emergency Medicine, Health Science University, Antalya Training and Research Hospital, Antalya, Turkey; II MD. Emergency Physician and Assistant Professor, Department of Emergency Medicine, Health Science University, Antalya Training and Research Hospital, Antalya, Turkey.; III MD. Emergency Physician, Department of Emergency Medicine, Health Science University, Antalya Training and Research Hospital, Antalya, Turkey.

**Keywords:** Ultrasonography, Granulocytes, Ischemic stroke, Echocardiographic measurement, Inflammation indicators, Acute cerebrovascular event

## Abstract

**BACKGROUND:**

Acute ischemic stroke (AIS) is the most common type of stroke. Inflammation is the primary factor in the pathogenesis of atherosclerosis. Use of immature granulocytes (IGs) has been recommended as a new indicator of systemic inflammation. However, data on the association between echocardiographic epicardial fat tissue thickness (EFT) and IGs in patients with AIS are limited.

**OBJECTIVE:**

To evaluate the association between the presences of IGs, epicardial fat tissue and AIS.

**DESIGN AND SETTING:**

Prospective study in a tertiary-care university hospital in Antalya, Turkey.

**METHODS:**

Our study included 53 AIS patients and 41 healthy controls with age and gender compatibility. Blood samples and transthoracic echocardiography of all participants were compared.

**RESULTS:**

IG levels were significantly higher in patients with AIS than in controls (0.62 ± 0.36 versus 0.28 ± 0.02, P < 0.001). The mean EFT was 3.74 ± 0.61 mm in the control group and 6.33 ± 1.47 mm in the AIS patient group. EFT was significantly greater in AIS patients than in controls (P < 0.001). For the optimum cut-off value for IG (0.95), the area under the curve (AUC) was determined to be 0.840; sensitivity was determined to be 81.1% and specificity, 92.5%. For the optimum cut-off value for EFT (4.95 mm), the AUC was determined to be 0.953; sensitivity was determined to be 90.6% and specificity, 90%.

**CONCLUSIONS:**

IG and echocardiographic EFT are clinical markers that can be used to predict AIS risk.

## INTRODUCTION

Stroke is a sudden localized and focal neurological syndrome. It is a major medical and economic problem that can result in severe disability and mortality. Acute ischemic stroke (AIS) is the most common type of stroke.^1^ Inflammation is the primary factor in the pathogenesis of atherosclerosis, and it is well known that inflammation indicators change before stroke and atherosclerotic events.^2^ In addition, many studies have shown that there are correlations between some inflammatory indicators such as high-sensitivity C-reactive protein (hs-CRP), neutrophil/lymphocyte ratio, cardiovascular diseases and AIS.^3,4^ Over recent years, use of immature granulocytes (IGs) has been recommended as a new indicator of systemic inflammation. The prognostic and predictive role of IGs has been shown in relation to many diseases.^5,6^ However, the role of IGs in patients with AIS has not been clearly revealed until now.

Epicardial fat tissue is located between the myocardium and the visceral sheets of the pericardium and it may play an adverse role in relation to the heart through production and secretion of proinflammatory and proatherogenic mediators. Its presence has been correlated with presence of atherosclerotic diseases such as coronary artery disease and aortic stenosis.^7-9^ Although many imaging methods are used for measuring epicardial fat tissue thickness (EFT), echocardiographic measurement is the method most preferred because of its inexpensiveness, repeatability and easy applicability. Although there are studies showing that EFT is associated with many diseases such as metabolic syndrome, diabetes mellitus and coronary artery disease, there are very few studies in the literature examining its association with AIS.^8-10^ Moreover, data on the association between echocardiographic EFT and IG in patients with AIS are limited.

## OBJECTIVES

The purpose of this study was to evaluate the association between the presences of IG, epicardial fat tissue and AIS.

## METHODS

### Study design and participants

Our study was designed as a single-center prospective case-control study. Its subjects consisted of patients who were admitted to our tertiary-care emergency department (ED) and were hospitalized with a diagnosis of stroke between December 2019 and December 2020. Our study was conducted in accordance with the Helsinki Declaration, upon approval from the local ethics committee (decision date: December 26, 2019; and number: 27/20). All participants were informed about the study before entering it, and a written consent statement was obtained from each of them.

### Data collection

Our study included 53 AIS patients and 41 healthy controls with age and gender compatibility. The individual medical history and clinical characteristics of all participants were recorded, and body mass index (BMI) was calculated as weight (kg)/height^2^ (m^2^). Patients were diagnosed with AIS through clinical and physical examination, radiology imaging and neurology consultation. All patients underwent brain tomography (CT) and/or diffusion-weighted magnetic resonance (MRI) imaging. Some patients were diagnosed with AIS through imaging with an appearance consistent with acute infarction, in repeated MRI imaging after hospitalization.

Patients with the following characteristics at the time of hospital admission were not included in the study: patients < 18 years old; obese patients (BMI > 30 kg/m^2^); pregnant women; patients with myeloproliferative disease; those with chronic inflammatory, chronic liver or kidney disease; those with malignancy; those with granulocyte colony stimulating factor, immunosuppurative agent or steroid use; those with uncontrolled hypertension or diabetes; and those presenting infection.

Blood samples were taken from peripheral venous blood within one hour at the latest, at the time of admission to the emergency department. All hemogram parameters, including IG, were measured using an automated blood analyzer (Coulter LH 780 Hematologic Analyzer, Beckman Coulter Inc., Brea, United States).

### Echocardiogram and epicardial fat measurement

Transthoracic echocardiography on all participants was performed in the left lateral recumbent position using a 2.5-3.5 MHz ultrasound probe (Mindray M5 device; Mindray DS USA Inc., Mahwah, New Jersey, United States). All sonographic examinations were performed at the bedside in the ED and were recorded to include three cardiac cycles. EFT was measured by an experienced emergency physician, and the guidelines of the American Echocardiography Society for standard echocardiographic measurements were followed.^11^ Measurements were made from the parasternal long axis.

**Figure 1.**
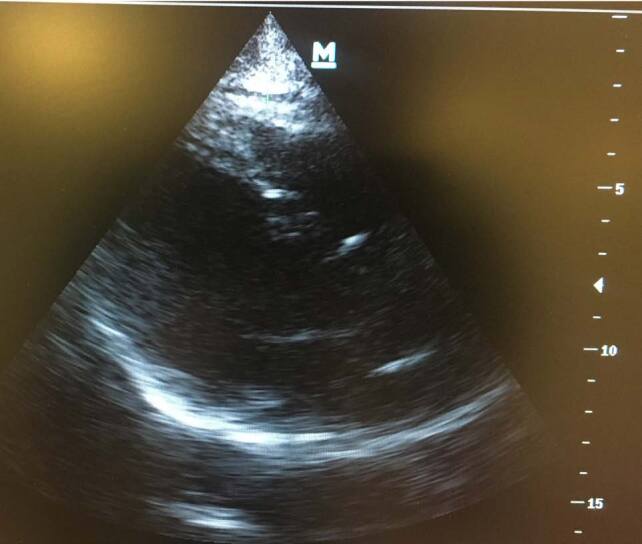
Measurement of epicardial fat tissue thickness by means of echocardiography.

In EFT measurements, the practitioner detected epicardial fat tissue as an area of relatively low echogenicity located between the right ventricle and the inner sheet of the pericardium. The greatest EFT in this area was measured in the end-systolic phase of the cardiac cycle, parallel to the aortic valve^12^ (Figure 1). Three consecutive measurements were made; the average was calculated and was noted on the patient’s follow-up paper as the EFT measurement. The investigator who made the measurements was blind to the laboratory values, final results and study group of the patients.

### Data analysis

Statistical analyses in our study were performed using the SPSS 21.0 software package (SPSS Inc., Chicago, Illinois, United States). Continuous variables were expressed as the mean ± standard deviation, and categorical variables as the number (%), for patients with AIS and the control group. The data were evaluated with regard to normal distribution, and the independent t test or Mann-Whitney U test was used, according to suitability. In addition, categorical data were evaluated using the chi-square test. Spearman’s correlation was calculated and examined to ascertain any correlations between the variables. The optimum cutoff value for IG and EFT, with regard to predicting AIS, was evaluated through receiver operating characteristic (ROC) analysis. Variables that might affect AIS were evaluated by means of logistic regression analysis. Statistical significance was defined as P < 0.05.

## RESULTS

**Figure 2.**
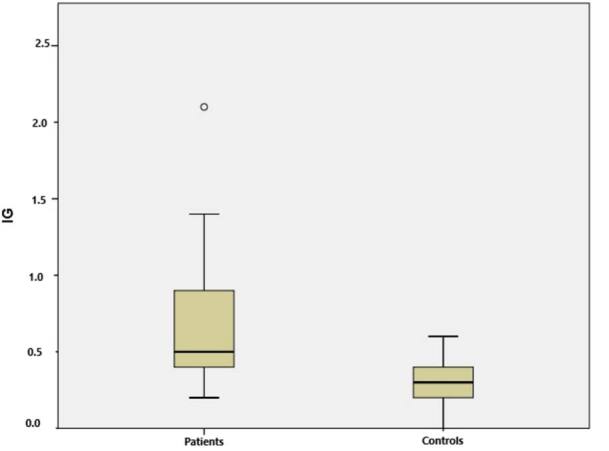
Box plot presentation of immature granulocytes (IG) in acute ischemic stroke (AIS) patients and healthy controls.

**Figure 3.**
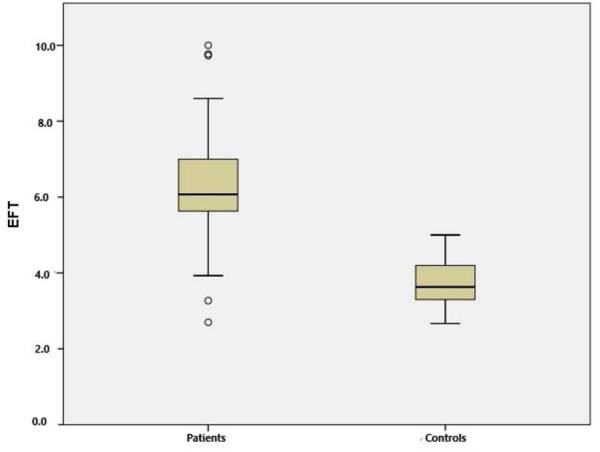
Box plot presentation of epicardial fat tissue thickness (EFT) in acute ischemic stroke (AIS) patients and healthy controls.

Among the 53 patients with AIS included in our study, 28 of them were male and 25 were female; their mean age was 71.15 ± 12.32 years. Among the 41 healthy participants, 19 of them were male and 22 were female; their mean age was 69.78 ± 10.31 years. There was no significant difference between the patient and control groups in terms of risk factors except for age, gender, BMI and hypertension (HT). The healthy control group had significantly lower systolic blood pressure, diastolic blood pressure, heart rate, leukocyte count and glucose level. IG levels were significantly higher in the patients with AIS than in the controls (0.62 ± 0.36 versus 0.28 ± 0.02; P < 0.001) (Figure 2). The mean EFT was 3.74 ± 0.61 mm in the control group and 6.33 ± 1.47 mm in the AIS patient group. EFT was significantly greater in the AIS patients than in the controls (P < 0.001) (Figure 3). The main characteristics of the patients are shown in Table 1.

**Table 1.** Baseline demographic characteristics of the study population

	Ischemic stroke (n = 53)	Controls (n = 41)	P-value
**Age in years, mean ± SD**	71.15 ± 12.32	69.78 ± 10.31	0.427
**Male, n (%)**	28 (59.6)	19 (40.4)	0.533
**SBP, mmHg (IQR)**	160 (40)	125 (22)	< 0.001
**DBP, mmHg (IQR)**	85 (26)	65 (13)	< 0.001
**Heart rate, beats/minute (IQR)**	89 (26)	75 (15)	< 0.001
**BMI in kg/m^2^, mean ± SD**	24.52 ± 2.99	25.30 ± 2.89	0.244
**EFT in mm, mean ± SD**	6.33 ± 1.47	3.74 ± 0.61	< 0.001
**Previous history, n (%)**			
Current smoker	7 (13.2)	10 (24.4)	0.162
Hypertension	36 (67.9)	17 (32.1)	0.010
Diabetes mellitus	20 (37.7)	13 (31.7)	0.544
History of CAD	24 (45.3)	23 (56.1)	0.298
**Laboratory findings, mean ± SD**			
WBC count (×10^3^/mm^3^)	9.18 ± 3.31	9.00 ± 2.84	0.967
Neutrophil (×10^3^/mm^3^)	6.41 ± 3.13	5.68 ± 2.61	0.215
Lymphocyte (×10^3^/mm^3^)	2.42 ± 0.41	2.34 ± 0.15	0.040
NLR	5.17 ± 0.93	3.11 ± 0.38	0.071
PLR	183.27 ± 27.72	133.68 ± 62.51	0.264
Hemoglobin, mg/dl	12.82 ± 2.12	13.18 ± 1.43	0.650
Glucose, mg/dl (IQR)	147 (75)	113 (22)	< 0.001
Blood urea nitrogen, mg/dl	22.13 ± 2.12	18.88 ± 6.64	0.506
Creatine, mg/dl	1.10 ± 0.69	0.91 ± 0.24	0.441
IG%	0.62 ± 0.36	0.28 ± 0.02	< 0.001
CRP, mg/dl	15.73 ± 4.96	6.21 ± 0.85	0.441
**Mortality, n (%)**	6 (11.3)	0 (0)	< 0.001

SD = standard deviation; SBP = systolic blood pressure; DBP = diastolic blood pressure; BMI = body mass index; EFT = epicardial fat tissue thickness; CAD = coronary artery disease; WBC = white blood cell; NLR = neutrophil-lymphocyte ratio; PLR = platelet-lymphocyte ratio; CRP = C-reactive protein; IG = immature granulocyte; IQR = interquartile range.

**Figure 4.**
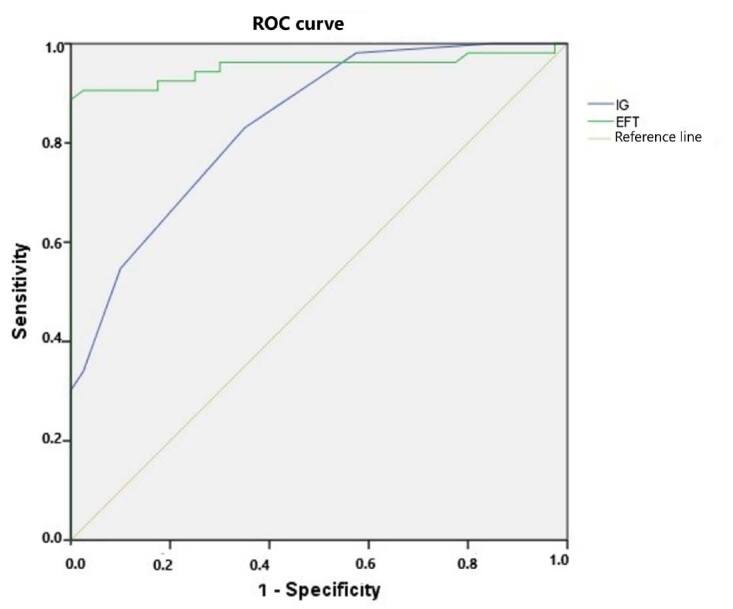
Receiver operating characteristic (ROC) curve analysis of immature granulocytes (IG) and epicardial fat tissue thickness (EFT) in predicting acute ischemic stroke (AIS) diagnosis.

The efficacy of IG and EFT for determining AIS was calculated by plotting ROC curves (Figure 4). For the optimum cutoff value of IG, which was 0.95, the area under the curve (AUC) was determined to be 0.840; sensitivity was determined to be 81.1% and specificity, 92.5% (Table 2). For the optimum cutoff value of EFT, which was 4.95 mm, the AUC was determined to be 0.953; sensitivity was determined to be 90.6% and specificity, 90% (Table 2). We also found a significant positive correlation between EFT and IG (r = 4.974; P < 0.001). We found that HT (odds ratio, OR: 2.990; 95% confidence interval, CI: 1.281 to 6.79; P = 0.011), high glucose (OR: 10.450; 95% CI: 3.935 to 27.749; P < 0.001), high IG (OR: 1.782; 95% CI: 1.624 to 1.937; P < 0.001), atrial fibrillation (OR: 1.612; 95% CI: 1.112 to 1.887; P = 0.008); and high EFT (OR: 2.733; 95% CI: 2.559 to 2.907; P < 0.001) were independent risk factors in multivariant logistics regression analysis (Table 3).

**Table 2.** Receiver operating characteristic curve analysis on the echocardiographic EFT and IG findings for prediction of acute ischemic stroke

Characteristics	Value	Sensitivity	Specificity	AUC (95% Cl)	P-value
IG	0.95	81.1	92.5	0.840 (0.807-0.901)	< 0.001
EFT	4.95	90.6	90	0.953 (0.926-0.974)	< 0.001

AUC = area under the curve; 95% CI = 95% confidence interval; IG = immature granulocytes; EFT = epicardial fat tissue thickness.

**Table 3.** Multivariate logistic regression analysis to assess predictors of ischemic stroke

Variables	OR	95% CI	P-value
Gender	1.297	0.573-2.936	0.217
Hypertension	2.990	1.281-6.79	0.011
Glucose	10.450	3.935-27.749	< 0.001
IG%	1.782	1.624-1.937	< 0.001
EFT (in mm)	2.733	2.559-2.907	< 0.001
Atrial fibrillation	1.612	1.112-1.887	0.008

OR = odds ratio; 95% CI = 95% confidence interval; IG = immature granulocytes; EFT = epicardial fat tissue thickness.

## DISCUSSION

This prospective study provides the first evidence in the literature demonstrating that increased EFT and greater IG are associated with AIS disease.

Epicardial fat tissue is a true tissue layer that accumulates around the heart and around the coronary vessels. It is fed through rich microcirculation since it contains neuronal network, stromal-vascular, immune and inflammatory cells.^13,14^

In recent studies, EFT has been shown to be associated with many cardiovascular and neurovascular diseases. Chu et al. showed that EFT was usable for predicting future cardiovascular events in patients with atrial fibrillation.^15^ Iacobellis et al. found high EFT values in patients with chronic atrial fibrillation and correlated this with development of heart failure.^16^ Wang et al. found significantly higher EFT values in patients who had gone through acute myocardial infarction, in comparison with a control group (5.6 ± 1.1 versus 4.1 ± 1.0 mm; P < 0.001). They also reported that high EFT values may indicate higher risk of mortality among patients with acute myocardial infarction.^17^ Tanɩndɩ et al. showed that patients with EFT values of 7 mm and above presented an association with death arising from cardiovascular events.^18^ Sagmacɩ et al. reported that there was frequently an association between high EFT levels and pain frequency, in a study conducted among patients with migraine.^19^ Akɩl et al. showed that mean EFT values in AIS patients were significantly higher than those of the control group in their study (5.95 ± 1.14 versus 4.86 ± 0.68; P < 0.001).^20^ In our study, we detected that EFT in patients with AIS was significantly greater than in the control group.

AIS is a condition associated with rapid loss of brain function that develops after a decrease in the blood supply to the brain. Atherosclerosis is a systemic disease that causes this process to develop. A number of cellular and molecular events associated with inflammation are known to contribute to development of atherosclerotic lesions and AIS. Therefore, increased vascular inflammation has been associated with AIS.^21,22^

In the literature, inflammatory biomarkers such as neutrophil to lymphocyte ratio (NLR) and platelet to lymphocyte ratio (PLR) have previously been used as measurements of correlations of leukocyte, neutrophil and lymphocyte counts with atherosclerotic processes and AIS.^23,24^ Over recent years, with technological developments, simple and easily measurable biomarkers such as IG have been used in relation to many diseases for diagnostic and prognostic purposes.

IG is not normally detected in peripheral blood in healthy individuals. However, it can become mixed with peripheral blood under inflammatory conditions.^25,26^ Use of this parameter has increased over recent years, although many doctors still do not consider it to be important. Its presence has been correlated with infection, sepsis and cardiovascular diseases. Moreover, its presence has been reported to be predictive of mortality in relation to many gastrointestinal diseases.^5,6,26^

No studies in the literature have yet examined the correlation between IG and AIS. Park et al. reported that IG might be a marker for mortality due to sepsis, and its sensitivity for mortality in their study was found to be 70%; specificity was found to be 78%.^27^ In a study conducted by Zeng et al., it was shown that there is a correlation between increasing IG values and positive blood cultures.^28^ In a recent study that we conducted, we found a correlation between increasing IG levels and positive indications for appendectomy.^29^ İncir et al. showed that use of IG together with hemogram indices enabled detection of inflammation and infection in the early period.^30^ In a study conducted by Karakulak et al., high IG values were shown to detect both disease severity and mortality in patients with acute pancreatitis.^31^ In our present study, we showed the correlation between increasing IG levels and AIS, for the first time in the literature.

Our study had some limitations. The first limitation was that even though our study was prospectively designed, it was conducted with only a small number of patients because of the coronavirus disease 19 (COVID-19) pandemic. Another limitation was that the EFT measurements of the patients included in our study were linear measurements, and our results could not be compared with the tomography and magnetic resonance imaging methods. Although echocardiographic measurements are dependent on the individual, they can be performed more easily and repeatably than can tomography and MRI. One of our major limitations was that the measurements were made by a single practitioner and also that these images were not interpreted by another researcher.

## CONCLUSION

This study showed the correlations between EFT, IG and AIS for the first time. IG and echocardiographic EFT are clinical markers that can be used to predict AIS risk.
